# Omega 3 Fatty Acids Promote Macrophage Reverse Cholesterol Transport in Hamster Fed High Fat Diet

**DOI:** 10.1371/journal.pone.0061109

**Published:** 2013-04-22

**Authors:** Fatima Kasbi Chadli, Hassane Nazih, Michel Krempf, Patrick Nguyen, Khadija Ouguerram

**Affiliations:** 1 INSERM, UMR 1087- CNRS UMR 6291, IRS – UN L'institut du thorax, Nantes, France; 2 UNAM Université, Oniris, Nutrition and Endocrinology Unit, National College of Veterinary Medicine, Food Science and Engineering, Nantes, France; 3 CRNH, Human Nutrition Research Center of Nantes, CHU, Nantes, France; 4 MMS 2160 Laboratoire de Biochimie, Faculté de Pharmacie, Université de Nantes, France; National Cancer Institute, United States of America

## Abstract

The aim of this study was to investigate macrophage reverse cholesterol transport (RCT) in hamster, a CETP-expressing species, fed omega 3 fatty acids (ω3PUFA) supplemented high fat diet (HFD). Three groups of hamsters (n = 6/group) were studied for 20 weeks: 1) control diet: Control, 2) HFD group: HF and 3) HFD group supplemented with ω3PUFA (EPA and DHA): HFω3. In vivo macrophage-to-feces RCT was assessed after an intraperitoneal injection of ^3^H-cholesterol-labelled hamster primary macrophages.

Compared to Control, HF presented significant (p<0.05) increase in body weight, plasma TG (p<0.01) and cholesterol (p<0.001) with an increase in VLDL TG and in VLDL and LDL cholesterol (p<0.001).

Compared to HF, HFω3 presented significant decrease in body weight. HFω3 showed less plasma TG (p<0.001) and cholesterol (p<0.001) related to a decrease in VLDL TG and HDL cholesterol respectively and higher LCAT activity (p<0.05) compared to HF. HFω3 showed a higher fecal bile acid excretion (p<0.05) compared to Control and HF groups and higher fecal cholesterol excretion (p<0.05) compared to HF. This increase was related to higher gene expression of ABCG5, ABCA1 and SR-B1 in HFω3 compared to Control and HF groups (<0.05) and in ABCG1 and CYP7A1 compared to HF group (p<0.05). A higher plasma efflux capacity was also measured in HFω3 using ^3^H- cholesterol labeled Fu5AH cells.

In conclusion, EPA and DHA supplementation improved macrophage to feces reverse cholesterol transport in hamster fed HFD. This change was related to the higher cholesterol and fecal bile acids excretion and to the activation of major genes involved in RCT.

## Introduction

Metabolic syndrome is a common pathological situation leading to an increase in cardiovascular disease. Dyslipidemia (higher triglyceride (TG) and lower HDL-cholesterol plasma concentrations) is frequently associated with metabolic syndrome [1]. Plasma HDL-cholesterol (HDL-c) levels are known to be inversely correlated with the risk of atherosclerotic cardiovascular diseases [1], however, this inverse relationship between HDL and cardiovascular disease reported in epidemiological studies is not confirmed in subgroups of patients with specific apoA-I mutations as ApoA1 Milano [2] or CETP polymorphism [3]. Then, a low plasma HDL-cholesterol concentration does not always predict an increase of the cardiovascular risk and deeper understanding of HDL metabolism could help to define the critical situations. The protective effects of HDL are mainly due to their central role in the reverse cholesterol transport (RCT), a process mediating the transport of cholesterol excess by HDL from peripheral tissues back to the liver for excretion into the bile and ultimately in the feces. In human, the cholesterol ester transfer protein (CETP) plays a critical role in RCT and performs, in parallel to direct uptake of HDL cholesterol by liver, transfer of cholesterol from HDL to LDL followed by liver LDL uptake. Thus, this metabolism is very complex and to study its modulation, it is easier to use animal models. Molecular mechanisms of RCT have been extensively studied in mouse [4], [5]. However, this animal model does not have any CETP and when it was over expressed in transgenic animals the rate of RCT was accelerated [6]. Therefore, CETP pathway would represent a major route for human RCT [7] and CETP expressing species, as hamster, represents a better model to investigate lipoprotein metabolism [8].

Omega-3 fatty acids such as docosahexaenoic acid (DHA) or eicosapentaenoic acid (EPA), abundant in fish oil, reduce clinical cardiovascular complications of atherosclerotic disease [9]. Several mechanisms have been proposed by which ω3PUFA reduce cardiovascular events, including triglyceride-lowering, anti-inflammatory, antithrombotic and anti-arrhythmic effects [10]. The effect of omega 3 fatty acid on reverse cholesterol transport has been tested in mice by Nichimoto et al, [5]. In this later study, authors showed that fish oil decreased HDL cholesterol and accelerated RCT by increasing excretion of HDL-derived ^3^H cholesterol recovered in fecal neutral sterols.

In our study, we investigated in hamster CETP species, whether omega 3 fatty acids supplemented high fat diet can modulate in vivo macrophage-to-feces RCT using hamster primary macrophages.

## Materials and Methods

### Ethics statement

All experiments were performed according to the regulations for animal welfare of the French Ministry of Food, Agriculture and Fisheries. The experimental protocol was adhered to European Union guidelines and was approved by the local Animal Used and Care Advisory Committee (Bretagne-Pays de la Loire committee). All animal trial was carried out under isofluran anesthesia. Animals were sacrificed by intra-cardiac injection of lethal dose of pentobarbital.

### Animal

18 males golden Syrian hamsters were obtained from Janvier (Le Genest-St-Isle, France) at 8 weeks of age weighting 80 to 90 g. They were housed in colony cages with wood litter (3 hamsters/cage) in controlled environment (22°C, 12/12 h light/dark cycle) and received water and diet ad libitum.

### Diets

Two high fat diet (HFD, 21% fat w/w), either enriched (HFω3) or not (HF) in ω3PUFA, and a control chow diet (5% fat w/w; Control) were used. Each of both high-fat diets were designed with the same composition and contained 38.7% starch, 24.7% proteins, 21% lipids, 8.5% minerals, 6% cellulose and 1.2% vitamins. The lipid mixture consisted of 15% lard, 3.5% palm oil, and 2.5% corn oil for HF and 1.5 g of lard was replaced with 1.5 g ω3PUFA oil mixture (Pierre Fabre Santé, Castres, France) for HFω3 diet. Both HF diets contained a low amount 0.014 g/100 g of cholesterol (from lard) and total lipids provided about 45% of total energy content. Control diet contained 23% protein, 58% starch, 5% lipids (2% corn oil and 3% palm oil), 8.5% minerals, 6% cellulose and 1.2% vitamins. 13% of energy intake was provided by lipids. Lipid composition of the 3 diets has been previously detailed [11] and contained respectively for Control, HF and HFω3 diets: SFA (39, 44.5 and 41%), MUFA (36, 46.5 and 41%), PUFA ω6 (24.5, 9 and 16.5%) and PUFA ω3 (0.5, 0.5 and 4.5%). For Control, HF and HFω3 diets, ω6/ω3 ratio was 50, 20 and 4 respectively. Experiments were performed in the three groups of animals at the end of a 20-week period of diet consumption.

### Biochemical analysis

After 20 weeks of diet, hamsters were fasted for 18 hours and blood was obtained by retro-orbital under isofluran anesthesia to determine plasma lipids. Plasma was then separated by centrifugation (4°C, 10 min, 3000 g). Total cholesterol and triglyceride were assayed using commercial kits (Biomerieux, France). Separation of HDL was performed using fast protein liquid chromatography (AKTA FPLC SYSTEM, GE Healthcare, USA). CETP activity was measured using a commercial kit (Roar Biomedical, NY, USA). Lecithin cholesterol acyl transferase (LCAT) activity was evaluated by measurement of free cholesterol content at T0 and after 1 hour of plasma incubation at 37°C. The decrease of free cholesterol concentration is expressed as the percentage of free cholesterol transformed into cholesteryl ester by hour [12].

### Isolation of peritoneal-elicited macrophages

Peritoneal macrophage were isolated as described in our previous study [13]. Hamsters were injected intraperitoneally (IP) with 15 mL of 3% Brewer thioglycollate medium (Sigma). Three days after injection, hamsters are sacrificed with lethal injection of pentobarbital and macrophages were collected by peritoneal washing with 10 mL ice-cold PBS. After centrifugation (1500 rpm for 5 minutes at 4°C), the pelleted macrophages were suspended in 5 mL of red blood cell lysis buffer (0.15 M NH4Cl, 10 mM KHCO3, EDTA 0.5 M) and incubated 15 minutes in ice. The macrophages were collected after centrifugation (1500 rpm for 5 minutes at 4°C) and suspended in RPMI culture medium supplemented with 10% FBS (Gibco). The cells were then plated in flasks in RPMI complete medium and allowed to adhere for 4 h in a 37°C humidified 5% CO_2_ incubator. Non adherent cells were removed with two washes of RPMI.

### In vivo macrophage-to-feces reverse cholesterol transport

Hamster macrophages were radiolabeled as described previously [13]. Briefly, 5 µCi/mL medium ^3^H-cholesterol and cholesterol loaded with 50 µg/mL acetylated LDL over 48 hours. Radiolabeled cells were then washed with RPMI and equilibrated for 4 hours in fresh RPMI supplemented with 0.2% BSA. Cells were pelleted by low speed centrifugation and suspended in PBS prior to injection into hamsters (n = 6/group). ^3^H-cholesterol–labeled and acetylated LDL-loaded cells (typically, 2.5 million of cells and 5×10^6^ cpm in 2.5 mL PBS) were injected IP into individually caged hamsters. Blood was collected via the retro-orbital sinus at 24, 48 and 72 hours and radioactivity in 20 µL of plasma was counted in a liquid scintillation counter. Hamsters were then sacrificed and liver was collected from each animal. Approximately 50 mg-piece of liver was homogenized in 400 µL water then lipids were extracted.

Fecal cholesterol and bile acid extraction was performed as previously described [13]. The total feces collected over 72 hours were weighed and soaked in Millipore water. One mL of the homogenized samples was used to extract the ^3^H-cholesterol and ^3^H-bile acid fractions. The extracts were evaporated, resuspended in toluene, and then radioactivity determined. Results were expressed as a percent of the radioactivity injected recovered in plasma, liver and feces (in cholesterol and bile acids fractions). The plasma volume was estimated as 3.5% of the body weight.

### In vitro measurement of cholesterol efflux

The ability of animals' serum to efflux cell unesterified cholesterol was measured by a procedure previously described [14] using [^3^H]-cholesterol–labeled Fu5AH. Briefly, Fu5AH was cultured in Eagle's minimum essential medium (EMEM) (Sigma, Saint-Quentin Fallavier, France) containing 10% fetal calf serum (FCS) (Sigma, Saint-Quentin Fallavier, France). Penicillin, streptomycin, and glutamine were present in all media. For efflux experiments, cells were plated in costar 24-well plates and grown in appropriate medium containing 10% FCS at 37°C in a humidified 5% CO2 atmosphere. When they were nearly confluent, cells were incubated for 24 hours at 37°C with 1 µCi/mL of [1,2-^3^H] cholesterol (10% FCS in EMEM). To ensure the label was evenly distributed among cellular pools, the labeling medium was replaced with EMEM containing 1% BSA (Sigma), and cells were incubated in albumin for 18 to 20 hours before measuring cholesterol efflux. The cells were then washed and incubated with the indicated serum prepared in EMEM (5% [vol/vol]), and efflux was performed for 4 hours. After efflux period, media were collected and counted for radioactivity by liquid scintillation counting. The residual radioactivity in the cells was determined after extraction with isopropanol. The results are expressed as ^3^H-cholesterol efflux into the culture medium as a percent of the initial ^3^H-cholesterol inside the cells.

### RNA extraction and gene expression analysis

Liver samples for mRNA analysis were homogenized, and RNA was isolated using Trizol reagent (Invitrogen, France). Real-time quantitative polymerase chain reaction (PCR) analysis was performed as follows: 1 µg of total RNA was reverse-transcribed using 100 units of MML-V reverse transcriptase (Promega, France). Real time quantitative PCR was performed on the 7000 Sequence Detection System with SYBR green MESAGREEN Master Mix Plus (Eurogentec, Angers, France). The reaction contained 10 ng of reverse-transcripted total RNA, 500 nM forward and reverse primers, 5× Sybr green Mix. Primers sequences are available on request. All reactions were performed at least in duplicate and Cyclophilin RNA amplification was used as a reference. Each couple of primers was tested in successive dilutions of cDNA to analyze and validate its efficiency. The expression of ABCA1, ATP binding cassette protein A1 (ABCA1), G1 (ABCG1), G5 (ABCG5), G8 (ABCG8); Cytochrome P450, family 7 , subfamily A, polypeptide 1 (CYP7A1), scavenger receptor class B type 1 (SR-B1), and low density lipoprotein receptor (LDLr) was measured (6 hamsters/group).

### Western blot analysis

For in vivo expression of SR-BI in hamster, proteins were harvested from liver of each group. The protein concentration was determined using the BCA Protein Assay method (Sigma Aldrich). For quantitative purposes, the same amount of protein (30 µg) was loaded for each liver sample. Proteins were separated on 4–15% SDS-PAGE and transferred to a nitrocellulose membrane. Membranes were blocked overnight with 5% milk TBS-Tween solution at 4°C, then washed and incubated for 2 h with primary polyclonal antibody against rabbit SR-BI (PA1-16803, Thermo Scientific). A second 2 h-incubation with a peroxidase-conjugated IgG secondary antibody was performed. Bands were visualized by Uptilight US Blot chemiluminescent substrate kit (Interchim). The abundance of β-actin was used as control (monoclonal antibody from Sigma Aldrich). Densitometric analyses of protein bands in the Western blots were done using G: Box Imager software (Syngene). Relative band intensities were derived by using the software to calculate integrated optical density for each band, and then each band was normalized with the integrated optical density of the corresponding β-actin band.

### Measurement of lipids content in liver

About 30 mg of hepatic tissue were used to extract lipid using Folch method [15]. Briefly the tissue was homogenized with chloroform/methanol (2/1), and then centrifuged to recover the liquid phase. The solvent was washed with 0.9% NaCl solution. After centrifugation the lower chloroform phase containing lipids was evaporated and lipids were suspended in 0.5 ml of ethanol. TG and cholesterol were then measured using Biomérieux kits reagents (TG: PAP 150, cholesterol RTU; Biomérieux, Marcy-l'Etoile, France) (n = 6/group).

### Statistical analysis

Results were expressed as means ± S.E.M. Statistical analyses were performed using Statview software (SAS Institute Inc., SAS campus drive, Cary, NC, USA). The ANOVA followed by PLSD Fisher's test was performed to estimate the effect of group and ω3PUFA supplementation (n = 6/group of hamster). Differences were considered significant at p<0.05.

## Results

### Effect of diets on body weight, plasma lipid parameters and LCAT and CETP activities (Table 1)

**Table 1:** Effect of different diets on body weight, plasma lipid parameters and CETP and LCAT activities.

	Control	HF	HFω3	ANOVA
Body weight (g)	120.0	134.3 *	123.8†	p<0.05
Plasma				
Triglyceride mmol/L	1.40±0.16	2.2097±0.19**	1.49±0.13	p<0.01
Total cholesterol mmol/L	3.35±0.08	4.02±0.06***	3.47±0.12†††	p<0.001
VLDL cholesterol mmol/L	0.209±0.005	0.581±0.008 ***	0.624±0.023***	p<0.0001
LDL cholesterol mmol/L	0.522±0.012	0.679±0.010***	0.681±0.025***	p<0.0001
HDL cholesterol mmol/L	2.621±0.064	2.760±0.042	2.097±0.077†††***	p<0.0001
LCAT activity (%/h)	20.28±1.19	17.19±1.0	21.02±1.5 †	p<0.05
CETP activity (pmol/h/µl)	60.1±4.1	61.3±4.01	52.6±4.11	NS

Values are mean ± SEM, n = 6 per group.

* different from Control (*p<0.05 ** p<0.01; ***p<0.001).

† different from HF († p<0.05, †† p<0.01; ††† p<0.001).

**Figure 1:**
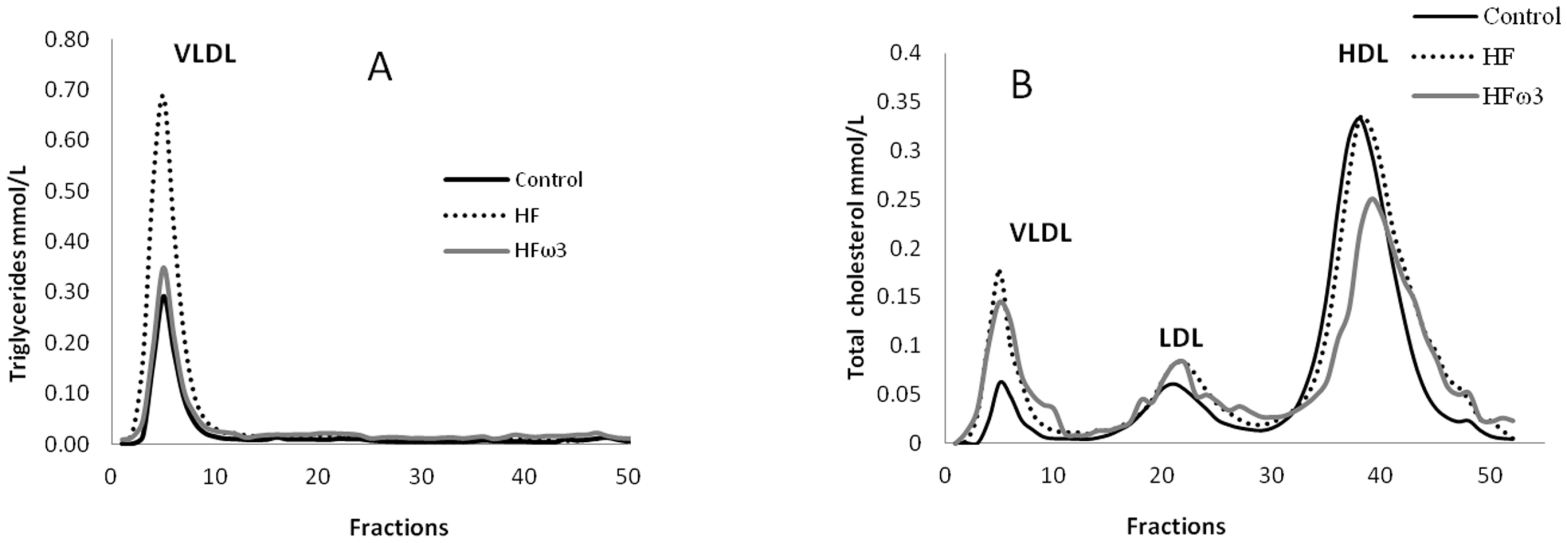
Representative TG (A) and cholesterol (B) profiles of Control, HF and HFω3 groups performed by FPLC.

HFD led to an increase of body weight compared to Control (12%, p<0.05), while HFω3 did not. Plasma and VLDL triglyceride (TG) were higher in HF group compared to Control (57%, p<0.01), related to higher TG content of VLDL (figure 1A), but lower in HFω3 compared to HF (46%, p<0.001). Cholesterol was higher in plasma (20%, p<0.001) and in VLDL and LDL (30%, p<0.001) in HF compared to Control, (figure 1B). Plasma total cholesterol did not differ in HFω3 compared to Control but significantly lower compared to HF (14%, p<0.001). This change was related to lower HDL cholesterol (24%, p<0.001) also obvious in figure 1B. LCAT activity did not differ between Control and HF and was higher in HFω3 compared to HF (22%, p<0.05). CETP activity did not differ among the three groups.

### Effect of diets on tissues lipids content

Liver TG content was higher in HF compared to Control group (14.2±2.1 mg/g *vs.* 8.5±1.1, *P*<0.05) and lower in HFω3 (11.1±2.6 mg/g) compared to HF group (*P*<0.05). No difference was observed in hepatic cholesterol content among the three groups (data not shown).

### Effect of diets on in vivo reverse cholesterol transport

**Figure 2:**
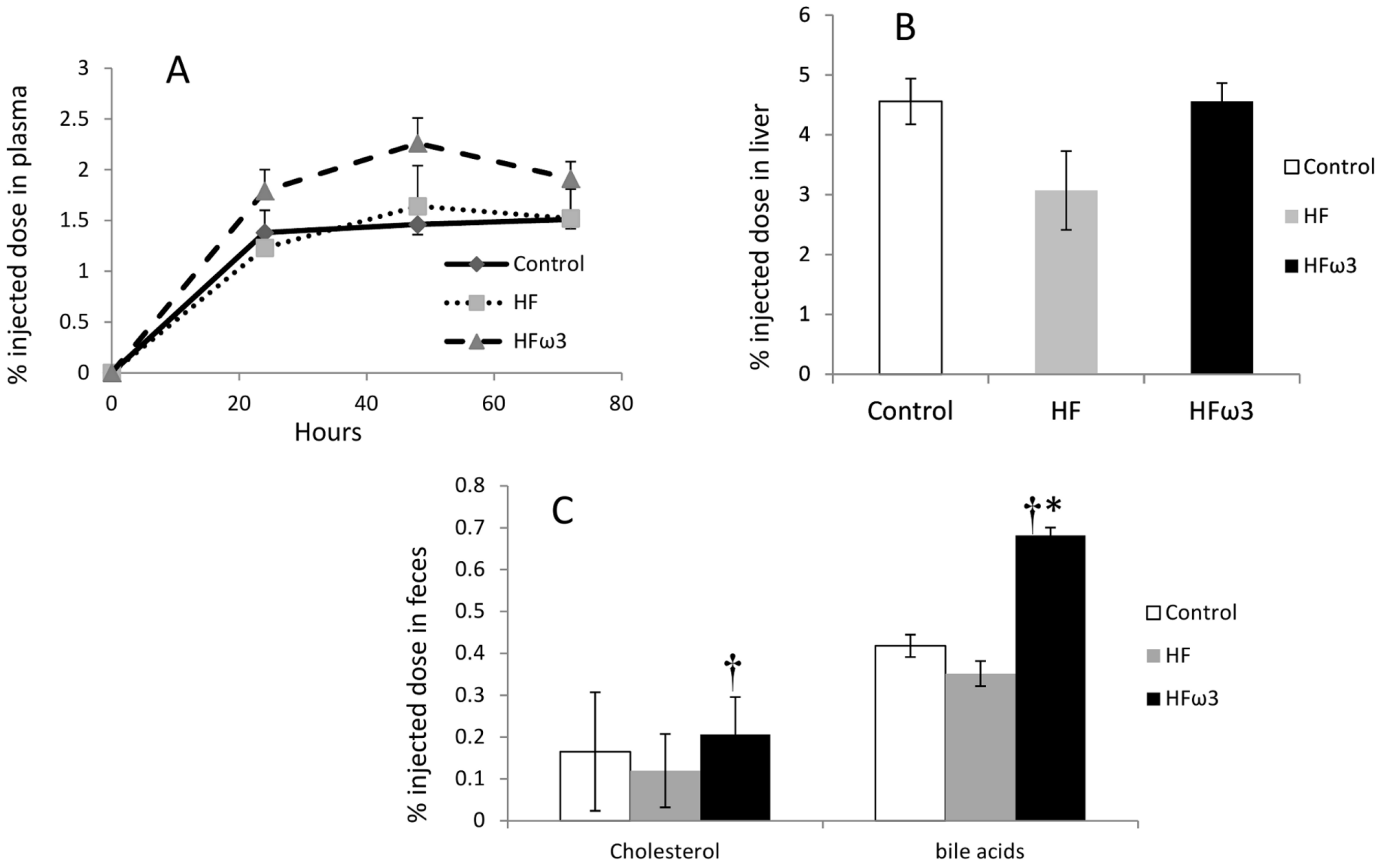
Effect of the three diets on reverse cholesterol transport. **A**: ^3^H-tracer appearance in plasma at time 24, 48 and 72 hours after injection. **B**: Liver ^3^H-tracer recovery at 72 h after injection of labeled and acetylated LDL-loaded macrophages. **C**: ^3^H-tracer recovery in fecal cholesterol and bile acids. Data are expressed as percent cpm injected and mean ± SEM (n = 6 per group; * p<0.05 different from Control; † p<0.05 different from HF.

To investigate whether reverse cholesterol transport is affected in vivo, Control, HF and HFω3 hamsters were injected with ^3^H-cholesterol labelled and cholesterol loaded hamster primary macrophages. As shown in figure 2A, plasma ^3^H-tracer appearance at 24, 40 and 72 h after macrophage injection did not differ among the three groups. No difference was showed in tracer recovery in liver among groups (Figure 2B). No significant change was observed between HF and Control for fecal cholesterol and bile acids excretion (Figure 2C). A higher ^3^H-tracer recovery was observed in fecal bile acids (60%, p<0.05) in HFω3 compared to Control. The tracer recovery in fecal bile acid (90%, p<0.05) and cholesterol (70%, p<0.05) was higher in HFω3 compared to HF.

### Effect of diets on in vitro reverse cholesterol efflux

**Figure 3:**
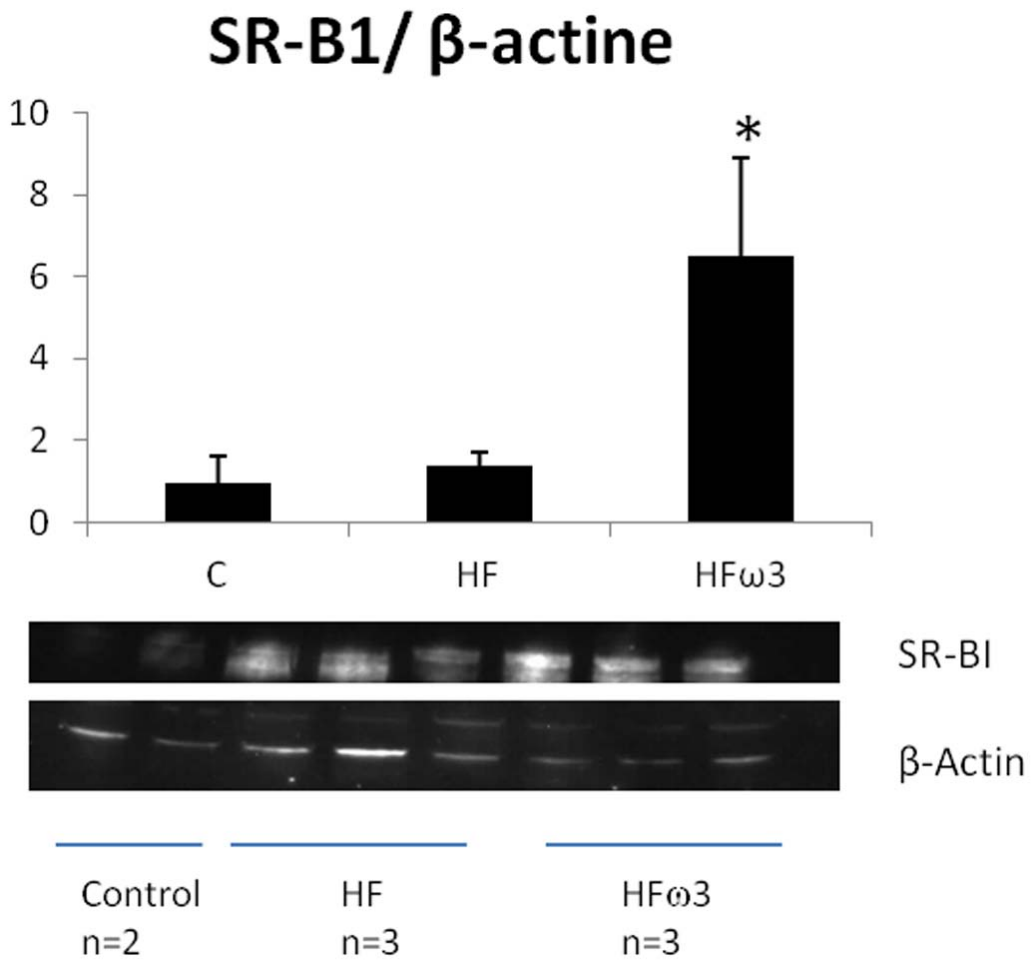
[^3^H]-cholesterol efflux from Fu5AH. [^3^H]-cholesterol labeled Fu5AH cells were incubated with hamster serum and efflux was performed for 4 h. Data are mean ± SEM. n = 6 per group.

We tested the ability of plasma from hamsters to promote cholesterol efflux from Fu5AH cells. After 4H incubation, HFω3 hamster plasma showed an increase in cholesterol efflux (30.99±1.99%) compared to HF and Control groups (24.57±0.42 and 25.79±1.48% respectively). No difference was observed between HF and Control group (figure 3).

### Effect of diets on genes involved in cholesterol metabolism

**Figure 4:**
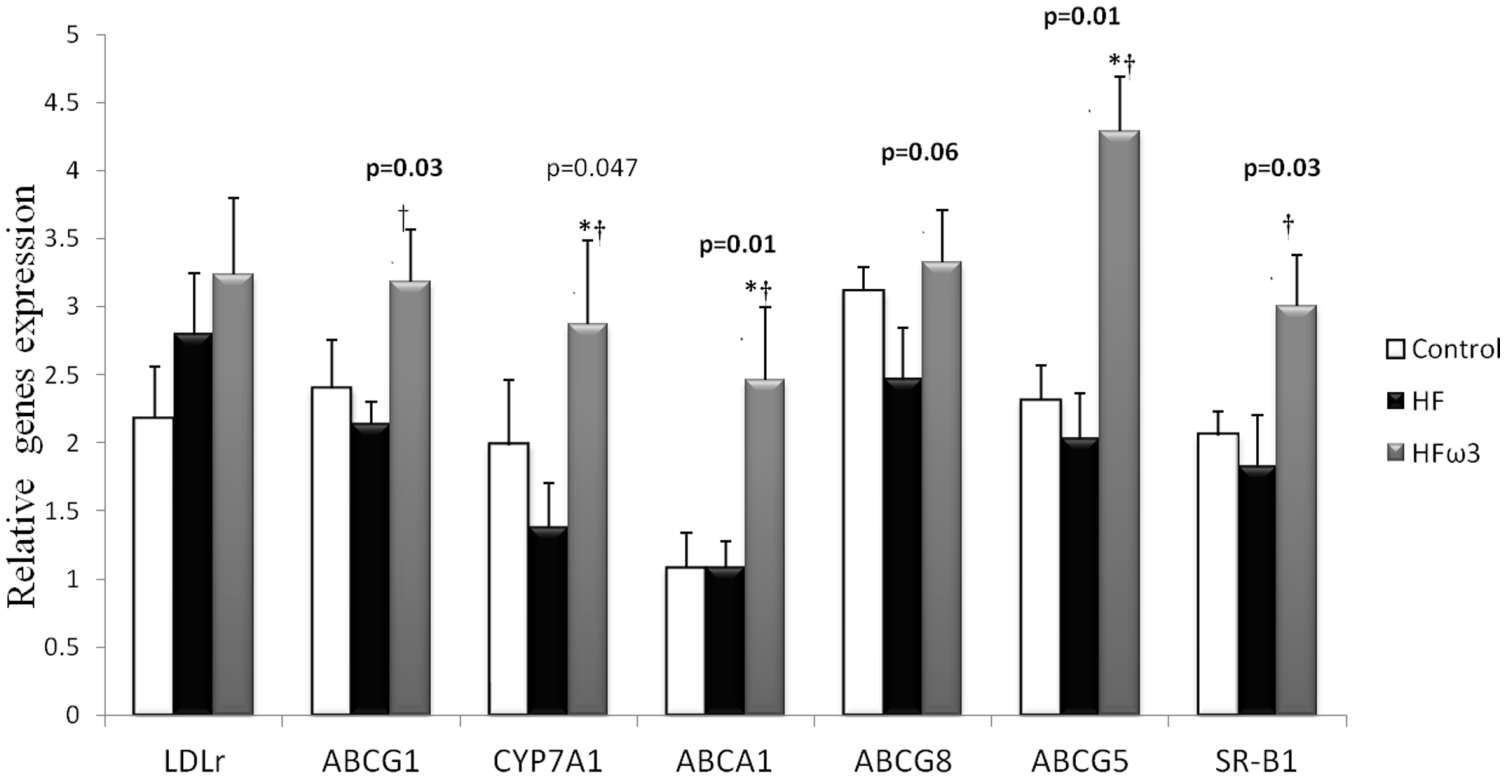
Effect of the three diets on hepatic gene expression. Relative gene expression in liver in Control, HF and HFω3 groups. Values are means ± SEM (n = 6 per group; * p<0.05 different from Control; † p<0.05 different from HF). (ABCA1, ATP binding cassette protein A1, CYP7A1, Cytochrome P450 family 7 subfamily A polypeptide 1, SR-B1, scavenger receptor class B type 1, and LDLr low density lipoprotein receptor).

Liver genes expression in the three groups is shown in figure 4. No change was observed for any studied gene between HF and Control groups. ω3PUFA supplemented high fat diet increased expression of the ABCA1 (127%, p<0.05), ABCG1 (49%, p<0.05), SR-B1 (65%, p<0.05), ABCG8 (35%, p = 0.06), ABCG5 (111%, p<0.05) and CYP7A1 (108%, p<0.05) compared to HF group. The ω3PUFA supplementation increased significantly the gene expression of CYP7A1, ABCA1 and ABCG5 compared to Control group. Expression of LDLr gene did remain unchanged among groups.

### Effect of diets on SR-B1 protein abundance

**Figure 5:**
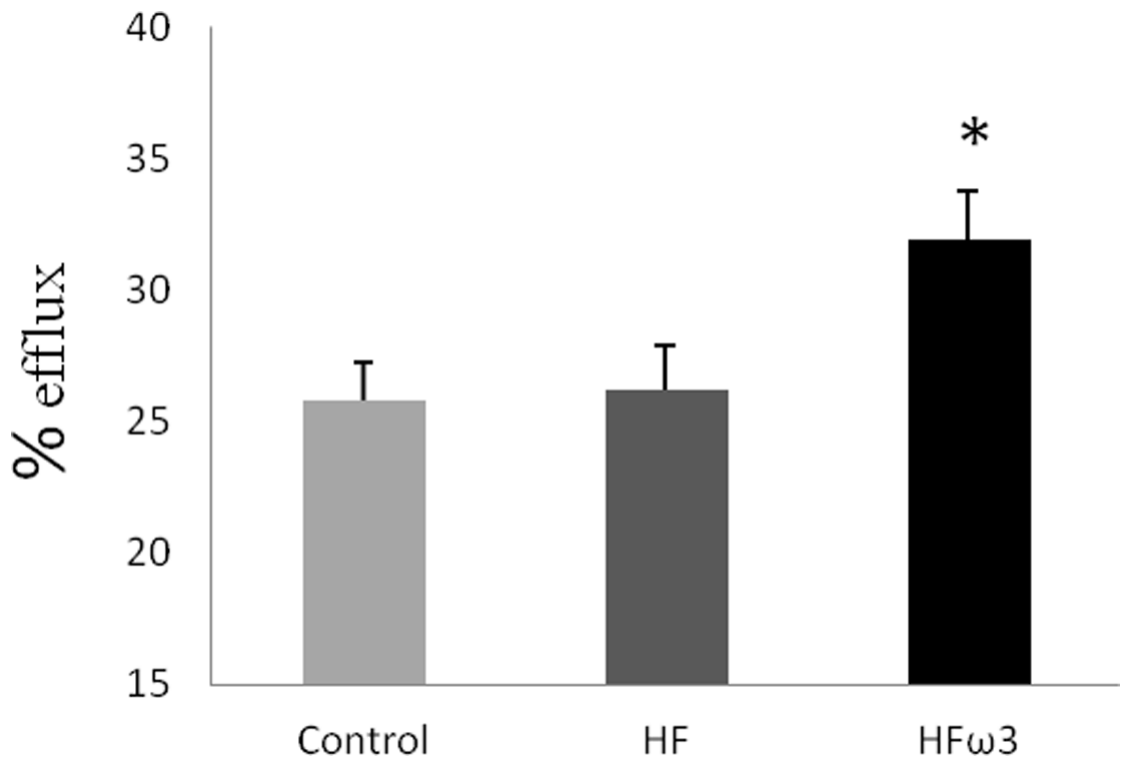
Western blot analysis of hepatic SR-B1 protein in hamster fed control, HF or HF diet supplemented with omega 3 fatty acids. Values are means ± SEM; n = 2 for Control group; n = 3 for HF and HFω3 groups.

Hepatic tissue SR-B1 protein abundance in FHω3 animals was significantly higher than that found in HF and Control groups (p<0.05) (figure 5).

## Discussion

In the present study performed in hamster, we showed that ω3PUFA prevented the increase of plasma TG and cholesterol by decreasing VLDL TG and HDL cholesterol concentrations respectively. These changes were related to increase of RCT efficiency as showed by a higher fecal bile acid and cholesterol elimination.

We have recently studied [13] the RCT in hamster using labelled primary hamster macrophages contrarily to more classical protocols using murine cells [16], [17]. This new protocol avoids the cross-species inflammatory reaction which could potentially lead to an immunological targeted destruction of macrophages when using J774 murine cells. Moreover, the J774 murine cells does not express endogenous apolipoprotein E (apoE) gene [18] involved in the removal of excess cholesterol from macrophage foam cells [19]. Hence, primary macrophages from hamster donors may be more reliable for assessing in vivo reverse cholesterol transport in hamsters [13]. But the probable pro-inflammatory reaction to this macrophage injection cannot be totally excluded.

In our study ω3PUFA supplementation had significant preventing effect against weight gain induced by high fat diet. The same results were obtained during ω3PUFA supplementation in rat [20] mice [21] and in hamster [11]. In this latter study, the expression of SCD1 decreased with omega 3 fatty acids supplementation. The inhibition of SCD1 is described to protect from HFD-induced obesity related to an up-regulation of genes involved in beta-oxidation and a down-regulation of lipogenesis gene expression as shown in mice SCD1−/− [22].

Comparing TG and cholesterol profile in HF and HFω3 groups, it can be established that ω3PUFA prevented the increase of plasma TG and cholesterol induced by high fat diet by decreasing VLDL TG and HDL cholesterol concentrations. A similar effect of ω3PUFA supplemented HFD on lipid profiles was previously reported in mice [23] and hamster [24]. Inversely, studies in rat [25] or in human [26] did not show any change in HDL cholesterol levels with ω3PUFA supplementation. This data divergence between studies could be mainly explained by difference in the diet (high or low fat, enriched or not in cholesterol). Improvement by ω3PUFA of plasma TG potentially disturbed by high fat diet is well known [27]. Such an improvement was also observed in our study concerning liver TG content. The normalization of VLDL TG concentration, potentially increased by production rate by high fat diet, suggests also normalization of VLDL TG production by liver. This improvement has been previously observed in hypertriglyceridemic human [28] or hamster [11] by ω3PUFA supplementation.

Cholesterol export from peripheral cells reduces intracellular cholesterol accumulation and atherosclerosis [29]. We reported that under ω3PUFA supplementation in vivo macrophage-to-feces reverse cholesterol transport was significantly accelerated, as shown by a significantly higher fecal cholesterol and bile acid excretion. This stimulated RCT was related to an increase in expression of most key genes involved in this process (ABCA1, ABCG1, SR-B1, ABCG5 and Cyp7A1). We therefore reported an enhancement of the ability of plasma from omega 3 fatty acid supplemented hamster to promote cholesterol efflux from Fu5AH cells. As in our study we have used Fu5AH for this assay, only the effect of omega 3 on the capacity of HDL to mobilize cholesterol from cells was measured. Similar to our findings, Montoya et al. found a positive effect of ω3PUFA rich diet on cholesterol efflux in-vitro using the same cell model [30]. Thus, the increased efflux measured is totally explained by the capacity of HDL from omega 3 supplemented diet to facilitate the cholesterol efflux. The HDL composition, not measured in our study, is probably affected by omega 3 fatty acids which known to influence HDL phospholipid acyl-chain composition [31], [32] and so cholesterol efflux capability [33], [34]. As mentioned higher, our essay did not allow to measure the capacity of macrophages to evacuate cholesterol but we cannot exclude a possible increase as shown by an enhanced ABCAI expression. ABCA1 and ABCG1 are key mediators of cholesterol efflux from macrophages to HDL particle, a first step of RCT in vivo. The fluxed cholesterol is then esterified in HDL by LCAT action which maintains this efflux [35]. We found an increase of ABCA1 and ABCG1 expression with ω3PUFA in good agreement with an increase of RCT and cholesterol efflux. Sheril et al. [36] reported in genetically dyslipidemic rat that ω3PUFA increase reverse cholesterol transport but with no change in ABCA1 expression. The same result was reported by Nishimoto et al. in mice [5] with no change in ABCA1 and ABCG1. Using apolipoprotein E-null mice, Zhang et al. described a decrease in the expression of ABCA1 and ABCG1 gene expression with a low ω6/ω3 ratio [37]. In vitro on murine macrophage cells, ω3PUFA regulate the activity of ABCA1 and ABCG1 by a mechanism involving LXR/RXR and alter cholesterol efflux [38]. Then, data on the effect of ω3PUFA on these receptors remains controversial related probably to different experimental conditions and animal or cell models. The scavenger receptor class B type I (SR-B1) plays an important role in meditating the uptake of HDL-derived cholesterol and cholesteryl ester in the liver. This receptor is also described as facilitating the initial step of HDL-mediated cholesterol efflux [39]. In our study, SR-B1was not measured in macrophage but its up-regulation in liver is described to promote macrophage RCT [40]. We reported here that ω3PUFA increased SR-B1 gene expression and protein abundance. In agreement with our study, omega 3 PUFA increased SR-B1 gene expression in hamster [24] and mice [23]. While Nishimoto et al. did not report any change in this gene expression [5]. Contradiction between these studies could be explained by the difference in the diet composition especially the amount of added cholesterol.

In our study we observed a higher LCAT activity in HFω3 in accord with a higher cholesterol efflux. In apoE−/− mice study [37], serum LCAT activity increased with decreased ω6/ω3 PUFA ratio. In human, alpha linolenic acid increase LCAT activity [41]. Conversely, Parks et al. have reported in monkey an inhibitory effect of dietary EPA and DHA on LCAT activity [42]. These inconsistencies could be related to the animal model or to the difference in composition of diets.

The stimulation of this initial RCT step was reported to be antiatherogenic [43]. It was also shown that dyslipidemia may contribute to the pathogenesis of mellitus diabetes type II. Loss-of-function mutations in ABCA1 show impaired insulin secretion [44] and increased cholesterol efflux improve insulin sensitivity [45]. Improvement of insulin sensitivity was observed in rats submitted to high fat diet enriched with ω3PUFA [46] and in our previous study [11].

The CETP activity did not change with omega 3 fatty acid supplemented diet. There are few studies on the effect of ω3PUFA on CETP activity. Data from literature are scarce and divergent with no change [47] increase [48] or a reduction [49] in CETP activity with ω3PUFA. Differences in the methods and diet used and the animal species studied may explain this discrepancies.

In the present study, hamsters fed ω3PUFA supplemented diet showed a higher cholesterol fecal excretion. The gene expression was significantly higher for ABCG5 and tended to be significantly higher for ABCG8. The same results were shown in mice [5] and rat [50], while in vitro study did not confirm these data [38].

The increase in cholesterol fecal excretion can also resulted from a decrease in intestinal absorption of cholesterol. NPC1L1 is indisputably one protein that plays a fundamental role in sterol absorption [51]. Nishimoto et al. reported an acceleration in macrophage to feces RCT with non-significant decrease in NPC1L1 gene expression [5]. However studies in hamster reported no effect or up-regulation on NPC1L1 gene expression with omega 3 fatty acids [52], [53] while in vitro studies showed a down regulation of this gene in Caco2 in the presence of omega 3 fatty acids [54], [55]. These divergent findings are not sufficient to conclude about the effect of omega 3 fatty acids on NPC1L1 modulation. As the implication of the intestinal absorption is not to be excluded in what we observed with omega 3 fatty acids, the effect of these fatty acids on cholesterol absorption must be investigated with specific method [56].

In our study, HFω3 showed a higher bile acids fecal excretion. This result was related to significantly higher gene expression of Cyp7A1. Cyp7A1 is the limited enzyme in bile acids synthesis and could be also involved in the increase of RCT. This effect was already reported in human [57] and in mice [58].

In the present study we reported a stimulated RCT by EPA and DHA as attested by an increased cholesterol and bile acids fecal excretion. As RCT is accomplished essentially by LDL and HDL cholesterol uptake by liver, the elevated cholesterol excretion in presence of ω3PUFA could be related to an increase in these two lipoproteins catabolism. But as the level of plasma LDL cholesterol and mRNA of LDLr gene did not change, any change in catabolism is unlikely. The measured increase in SR-B1 expression suggested an increase in the catabolism of the HDL which could explain a decrease of their concentration. Then our results suggest that, although conditions providing increased HDL cholesterol are generally required to prevent cardiovascular disease, decrease of HDL cholesterol as a consequence of stimulated RCT is atheroprotective index.

In conclusion, dietary ω3PUFA supplementation in high fat diet fed hamsters improved body weight, plasma TG and cholesterol and promoted excretion of cholesterol and bile acid into the feces through RCT. This probably results of an increase in the efflux of cholesterol from peripheral tissue to HDL particle, due to the activation of ABCA1 and ABCG1, and an increase of cholesterol movements from HDL into feces due to the activation of SR-B1, ABCG5 and CYP7A1. Our results may contribute to explain the anti-atherogenic effect of ω3PUFA and also underline the complexity of considering HDL cholesterol concentration as atheroprotective index.
